# The Motor Function Neurological Assessment (MFNU) as an indicator of motor function problems in boys with ADHD

**DOI:** 10.1186/1744-9081-5-22

**Published:** 2009-05-18

**Authors:** Liv Larsen Stray, Torstein Stray, Synnøve Iversen, Anne Ruud, Bjørn Ellertsen, Finn Egil Tønnessen

**Affiliations:** 1 Department of Child and Adolescent Health, Sørlandet Hospital, Norway; 2 The Reading Centre, Faculty of Education and Arts, University of Stavanger, Norway

## Abstract

**Background:**

The paper presents the Motor Function Neurological Assessment (MFNU), as a tool for identifying typical motor function problems in children with Attention Deficit Hyperactivity Disorder (ADHD). The study investigated motor functions in boys diagnosed with Hyperkinetic Disorder (HKD, F.90.0). HKD corresponds to the ADHD-combined (ADHD-C) diagnosis in the DSM-IV. The paper addresses the ability of the instrument to discriminate between non-medicated boys with HKD and a control group consisting of normal non-referred boys without any clinical significant ADHD symptoms.

**Methods:**

25 drug-naïve boys, aged 8–12 years and recently diagnosed as HKD F90.0, were compared with 27 controls, all boys in the same age range, on 17 MFNU subtests, and with a 'Total score' parameter.

**Results:**

On the individual subtests 80–96% (median 88%) of the ADHD group showed 'moderate' to 'severe' problems, compared to 0–44% (median 14.8%) within the control group. The percentage of 'severe problems' ranged from 44–84%, (median 64%) in the ADHD group, and 0–44% (median 0%) in the control group. The highly significant differences found between the groups on all subtests, and on the Total score scores, indicated that the MFNU had a high discriminative power when children with ADHD and normal controls were compared. The Total score parameter seemed to be a meaningful discriminator of a common underlying factor of the 17 subtests used in the study.

**Conclusion:**

The study confirms our clinical findings that the MFNU measures a consistent pattern of motor function problems in children with HKD, and that these problems are rarely represented in individuals without ADHD. Further research is needed to investigate to what extent the MFNU taps motor problems that are truly specific to ADHD, in contrast to motor problems common to children with DCD or other clinical problems.

## Background

The Motor Function Neurological Assessment (abbreviated MFNU in the Norwegian edition of 'Motorisk Funksjonsnevrologisk Undersøkelse') has been developed over a 25 year period based on clinical observations and assessments of children referred for evaluation of possible Attention Deficit Hyperactivity Disorder (ADHD). The individual subtests were for a large part based on a wide representation of established tests of motor functions in children [1]. The selection criterion for inclusion of subtests in the MFNU was that the majority of children with clinically significant behavioral ADHD symptoms obtained a problem score on the subtest. In its present form, at the time of the study, the MFNU consisted of 18 subtests. The main motivation for developing the tool was to demonstrate motor function problems in children with ADHD to parents and teachers in a systematic way, and to show how these problems might affect activities of daily life and interaction with others negatively.

When parents and teachers were asked to describe the individual children problems besides ADHD symptoms, they rarely mentioned problems with motor skills. On the contrary, motor skills were very often rated as major assets. Our experience is in accordance with Fliers et al. [2]. They found that about one third of parents and teachers of children with ADHD reported the child to have motor problems. Standardized assessment tools like the quantitative part of the Movement Assessment Battery for Children (M-ABC) [3] and neuropsychological tests i.e. the Halstead-Reitan Test Battery [4] seemed to confirm this impression, showing limited or no motor impairment for many of the children. However, through our observations in natural settings the children with ADHD almost invariably were struggling with tasks requiring fine muscular adjustments. These problems seemed to affect many areas of functioning, for instance fine motor adjustments involved in eating routines and in hand-writing [5]. Poor fine motor coordination in children with ADHD are well known [6]. Our observations, however, revealed motor problems associated not only with fine motor adjustment, but also with regulation of gross movements e.g. synkinesis [7], and with the stabilization of the trunk. The children made a mess while eating, and were frequently and involuntarily bumping into things. Many had problems performing certain athletic sports [6], lacking bounce when walking and running, and became more easily tired and exhausted than peers. At school we observed that the children often rested their upper body on the desk, supporting the head, or would frequently slide down on the chair into a leaning position. The children were often described by parents and teachers as being distracted, unmotivated, disobedient, aggressive, destructive or uncooperative. However, careful clinical observations and testing of motor functions indicated that behaviour can be understood otherwise. At least part of their "disobedient" and "oppositional" behaviour could be better understood as problems associated with poor motor control and movement coordination [8].

A close examination of the specific motor tasks involved in the MFNU subtests indicated that the children had problems with motor inhibition. The children with ADHD also displayed increased muscle tone in the gross movement muscles (e.g. m. Iliopsoas and m. Latissimus dorsi) restricting movements of shoulders, arms and thorax. In most cases we found little or no training effect even after prolonged training periods focusing on the special tasks involved in the subtests. We also observed marked improvements in performance on the MFNU 1 1/2 hour after administration of central stimulants (methylphenidate, MPH). When the physiological effect of the stimulant subsided, the motor problems returned undiminished. We observed this pattern even in individuals who had been medicated with MPH for many years [9]. An example of motor inhibition problems and the effect of MPH, is illustrated by the videos of the subtest 'Thumb movement' without medication (see Additional File 1) and 1 1/2 hour later after medication with 10 mg MPH (see Additional File 2). The child in the video has been medicated with MPH for several years, and was taken off medication a day before the recording.

### ADHD, hyperkinetic disorders and motor problems

The definition of ADHD used in Norway, and in our work, is based on the International Classification of Diseases (ICD-10) diagnosis hyperkinetic disorders (HKD, F90). HKD usually arises in the first 5 years of life. Six or more symptoms of inattention, three or more symptoms of hyperactivity and one or more symptoms of impulsiveness are necessary for the diagnosis [10,11]. The American Psychiatric Association's Diagnostic and Statistic Manual (DSM IV) [12] identifies three subtypes of ADHD: ADHD-predominantly inattentive (ADHD-PI), predominantly hyperactive-impulsive (ADHD-HI), and ADHD combined (ADHD-C). Only persons with ADHD-C will meet the HKD criteria. Due to the more restrictive inclusion and exclusion criteria of the HKD diagnosis in comparison to the DSM IV ADHD diagnosis the incidence rate is much lower for HKD than for ADHD. It is about 1–2% for HKD, compared to 5–10% for ADHD [10]. Lahey et al. [13] found that 26% of younger children with ADHD met the criteria for HKD. This fact makes a direct comparison of the two diagnoses difficult.

In its definition of HKD the ICD-10 states that: "Impairment of cognitive functions is common, and specific delays in motor and language development are disproportionately frequent." [11]. There is currently no acknowledgment of motor problems within the differential diagnosis section for ADHD in the DSM-IV [12]. It seems to be assumed that the motor problems seen in ADHD either are separate, co-morbid conditions [6] or side effects of dysfunctional attention or impulsiveness. The differential diagnosis section for the DSM-IV diagnosis 'Developmental Coordination Disorder' (DCD), states that "Individuals with Attention-Deficit-Hyperactivity Disorder may fall, bump into things, or knock things over, but this is usually due to distractibility and impulsiveness, rather than to a motor impairment" [12]. While much of the "clumsiness" observed in children with ADHD may be interpreted as an expression of attention problems, or general impulsivity, it is widely recognized, however, that many children with ADHD have motor impairments [6,7,14-19].

Pitcher et al. [18] have demonstrated that poor fine motor ability found in children with ADHD could not be attributed to deficits in attention or concentration, but rather to factors relating to their motor ability. Similar findings are reported by Miyahara et al. [20]. Denckla and Rudel [7], using discriminant function analyses for speed, rhythm, and overflow correctly classified 89% of the boys as those with "hyperactive" versus "normal" behavioral histories. They concluded that neurological examination of "hyperactive" boys does reveal developmentally immature coordination. Uslu et al. [21] found the factor 'speed of movement', which contained items related to cerebral coordination of alternate muscle groups, to differ significantly between children with ADHD only and children with learning disorder (LD) or with ADHD-LD comorbidity. Kalff and collaborators [22] found that children at risk for ADHD were in general less accurate and more variable in their movements than children with other psychopathology and controls. Children with ADHD performed jerky movements and required more time than controls to change the direction of movement [23,24]. Raberger and Wimmer [25] showed that children with ADHD not only have fine motor problems, but also impaired balance.

An overlap of 30–50% has been reported between ADHD and the DCD diagnosis [18,26,27]. DCD is defined as a marked impairment in the development of motor coordination that significantly interferes with activities of daily living, and is not related to a medical condition [12]. The standardized motor assessment battery M-ABC test [3], which addresses the areas manual dexterity, ball skills and balance, is commonly used in the diagnosis of DCD in studies assessing motor problems in ADHD [17,28,29]. It is quite possible that ADHD and DCD might coexist as separate disorders, and that the M-ABC test in an effective way reveals cases of comorbid ADHD and DCD. However, our clinical experiences are that children with ADHD may display motor problems in natural settings that are not uncovered using this test. A child with a high tone in m. Latissimus dorsi may obtain no problem scores on the ball items in the M-ABC test, but a restriction of the shoulder movement is often revealed by the MFNU when the child throws a ball with the arm in an upwards position. Neuropsychological tests like The Grooved Pegboard [30], the Maze coordination task [30], and the Finger-tapping test [4] yielded results similar to the M-ABC test [31].

Rasmussen and Gillberg [32] found that motor problems often persist into adulthood for persons with ADHD. Meyer and Sagvolden [16] found significantly poorer performance within the 3 ADHD groups, one for each subset of the DSM-IV diagnosis, compared to a control group of normal children on the Grooved Pegboard Test and Maze Coordination Task. Problems with motor control were less noticeable in the older groups. However, the authors argue that the differences found probably were due to the effect of maturation, which made the tasks too easy for this particular age group. No effects of gender were found in this study. In a retrospective study of 73 children with ADHD age 5–17 years (62 boys and 11 girls) assessed by items of MFNU, Stray [1] found that motor problem were present both in the younger ADHD group (age 5–10) and in the older group (age 11–17). There were no significant age effects on motor performance on any of the subtests neither in the medicine responder group nor in the non responder group. Gender differences in performance were not found.

### The neurofunctional problems of ADHD – possible relations to motor problems assessed with the MFNU

Stray [1] and Stray et al. [9] hypothesized that there may be a functional relationship between the behavioral symptoms of ADHD and motor problems, which are not accounted for by attention deficits/impulsivity or comorbidity (DCD). Children with ADHD appear to have prefrontal mediated dysfunctions resulting in difficulties with impulse/inhibition control and self-regulation associated with higher order executive functions [33-35]. Several studies using the stop-signal task have shown a poorer inhibition performance and longer stop-signal reaction time in children with ADHD compared with a normal control group [36,37].

#### 1. Motor inhibition

Motor inhibition problems typically reported in the ADHD literature are mainly associated with higher order executive functions like motor planning, timing and evaluation [33]. Our clinical findings do, however, suggest that disinhibition is involved also at a more basic motor function level, accounting for the fact that children with ADHD often appear clumsy and uncoordinated. This is also reported in the literature. Deeper brain areas closely associated with motor control seem to be involved in ADHD, particularly the frontostriatal system and basal ganglia [16,38,39]. Sagvolden et al. [40] state that altered dopaminergic function in ADHD, hypofunctioning mesocortical and nigrostriatal dopamine branches will give rise to development of hyperactivity in novel situations, impulsiveness, deficient sustained attention, increased behavioral variability, disinhibition, clumsiness and "neurological soft signs". Dopamine acts as a key neurotransmitter in the brain. Numerous studies have shown its regulatory role in motor functions [41].

Altered brain activity is found in the right inferior frontal cortex, left sensorimotor cortex, and bilateral cerebellum and the vermis as well as in the right anterior cingulated cortex, left sensorimotor cortex, and bilateral brainstem in children with ADHD during resting-state [42]. Berquin et al. [43] showed a reduced volume of cerebellar vermis in boys with ADHD. They linked deficits in motor inhibition seen in ADHD to dysfunctions in the cerebellum. Similar findings are reported in girls with ADHD [44]. The cerebellar connections to frontal motor areas enable the cerebellum to improve motor skills, in the same manner as they seem to be involved in mental and language skills [45]. Cerebellum is involved in the coordination of movement, particularly the ability to rapidly conduct successive movements. Pronation/supination of the hands (diadokokinesis) is often used to assess coordination of movement in a patient suspected of having a cerebellar dysfunction [46].

Problems with motor inhibition are observed with the MFNU both at the fine and gross motor level through varying motor tasks performed by the child, and in examinations of passive movements. A typical characteristic is increased muscular tone when flexion-extension movements are repeated several times in succession, resulting in a restricted movement range and in jerkiness of the movement. Such a restriction can be illustrated by the subtest 'Thumb movement' (see Table 1 and the video Additional File 1). This subtest reveals an increasing stiffness in the thenar muscles in children with ADHD when finger opposition followed by abduction/extension movements of the thumb is repetitively performed. After administration of I0 mg MPH, the 'Thumb movement' is performed without restriction/heightened tone in the muscles (see video Additional File 2)

**Table 1:** The subtests of MFNA used in the study

Name of subtests and video examples (a girl without motor problems)	Description
01. Dynamic balance-2 legs (see Additional File 3)	Three sideway jumps within marked squares, back and forth. The entire process is repeated three times without stopping.
02. Dynamic balance-1 leg (see Additional File 4)	Three sideway jumps on one leg within marked squares, back and forth. The entire process is repeated three times without stopping. Both legs are tested.
03. Diadochokinesis-right 04. Diadochokinesis-left (see Additional File 5)	Pronation-supination of one hand, the elbow flexed 90 degrees. The hand is held as an "extension" of the lower arm. The exercise is performed for approximately 15–20 seconds.
05. Reciprocal coordination (see Additional File 6)	Alternate clenching of one fist, and stretching of the other in a rhythmic manner, for about 15 seconds. Fingers should be nearly completely extended after the hand has been clenched. Elbows at a 90 degree angle, palms facing upwards.
06. Thumb movement (see Additional File 7)	The tip of the other fingers are successively touched with the palmar surface of the tip of the thumb. After each opposition the child extends and abducts the thumb. Both hands are tested for approximately 20 seconds.
07. Throw ball (see Additional File 8)	The tester plays ball with the child. A fairly large ball is used. The child has to throw with dominant arm in an upwards position. Shoulder movement is scored.
08. Catch ball (see Additional File 9)	The tester plays ball with the child. A tennis ball is used. The child has to catch the ball with one hand, fingers flexed, without touching the body.
09. Walking (see Additional File 10)	Walking with toes alternately pointing outwards ("Chaplin") and inwards, followed by walking on the outer foot rend (Fog's test) and inner foot rend.
10. Lifting arm (see Additional Files 11)	Lies prone, arms in a 45 degree angle from midline, lifting one arm with the palm of the hand facing the floor.
11. Lifting leg (see Additional Files 12)	Lies prone, spina iliaca anterior is touching the floor while lifting one stretched leg at a time.
12. "Flying" (see Additional Files 13)	Lies prone, the arm in a 45 degree angle from midline, lifting head, arms and legs.
13. Passive abduction-right hip 14. Passive abduction- left hip (see Additional Files 14)	Lies supine. Tester holds the child's knee and hip in a flexed position. The tester stretches and flexes the leg to elicit a relaxation of the hip muscles, and abducts the leg. The sides are evaluated separately.
15. Passive movement-right foot 16. Passive movement-left foot (see Additional files 15)	Lies supine. Tester examines passive movement with dorsal flexion and eversion/plantar flexion of the right and the left foot.
17. Synkinesis (see Additional file 16)	'Synkinesis' is not a separate test, but an item for the evaluation of synkinetic movements registered in one or more subtests. When observed, the tester tries to correct it. The remaining synkinesis after correction is scored.

Other levels of motor inhibition problems are observed as difficulties stopping and changing the direction of the movement when jumping [9], and as presence of synkinesis (overflow of movements). Overflow movements in ADHD are well known [7,47,48]. Age-inappropriate overflow may reflect immaturity of cortical systems involved in automatic inhibition [47].

#### 2. Problems in proximal stabilization

Studies have shown that children with ADHD have balance problems [17,25]. Our own clinical observations indicate that balance problems in ADHD may be linked to difficulties in keeping the trunk in an erect position using the proximal stabilizing muscles of the column. In our work we have almost invariably observed that children with ADHD display a high muscle tone in the gross movement muscles, especially the m. Sacrospinalis, m. Latissimus dorsi and in m. Psoas major [9]. The high muscle tone may have many possible explanations. One of them is linked to a hypothesis of an excessive use of gross movement muscles to compensate for a lack of proximal stabilization. Compensatory use of musculature is well known [49]. Use of the gross movement muscle groups in stabilization of the trunk might eventually lead to a restriction of the natural movement of shoulder and hip, and of the thorax. The result may be observed as stiffness, tiredness, bodily restlessness, restricted breathing and in the need to support/rest/vary the position of the trunk e.g. while sitting for prolonged periods.

Why would children with ADHD have problems with proximal stabilization? Regulation of the central nervous activation (level of arousal) is partly connected to the reticular formation in the brainstem [50]. Stray [1] and Stray et al. [9] argue that activation problems related to the reticular formation reported in ADHD [51,52] may negatively affect the regulation and the tone of the deep stabilizing muscles of the column. This may result in a need to use compensatory muscles to maintain body stabilization. According to Brodal [53] muscle tone may be increased or decreased based on the balanced influence from inhibitory and facilitating regions of the reticular formation. Reticulospinal fibres particularly connect to motor neurons in the spinal cord which affect proximal extremity muscles and muscles that stabilize the column. These fibers are of particular importance for the maintenance of upright posture, orientation of the body and head towards objects in the surroundings and for certain grosser movements of the extremities [53]. The hypothesis promoted by Stray [1] suggests that there may be a functional link between the regulation of muscular tone and activation. An implication of this hypothesis would be an expected simultaneous improvement in the regulation of activation and in muscle tone by the introduction of central stimulants to persons with ADHD. This issue is addressed in another article under preparation covering the second part of our study.

### The aim of the present study

Through the development period of the MFNU [1] motor inhibition and balance problems showed up as the clinically most significant aspects of motor problems observed in children who were later received the ADHD diagnosis. The study performed by Stray [1] indicated a close link between positive response to central stimulants and a high total problem score on the MFNU, suggesting that problems with motor inhibition and stabilisation of the trunk were functionally linked to ADHD. A two-part study was planned to investigate this issue in a controlled manner. The present article represents the first part of this study. Our aim has been to investigate the ability of the MFNU to discriminate between children with ADHD-C/HKD and a control group without behavioral symptoms of the condition.

Our research questions were:

To what extent do children with ADHD-C/HKD show motor problems, as measured by the MFNU, and how well does the test discriminate between children with ADHD and normal controls? We hypothesized that children with ADHD-C/HKD would display consistently high problem scores, and show significantly more motor problems on all the subtests of the MFNU, used in this study, compared to children without ADHD.

## Method

### Sample

Fifty-two boys, all Caucasian Norwegians, participated in the study. Informed consent was given by the parents. Twenty-five drug naïve boys, aged 8–12 (mean 10.2 years, SD 1.3), out-patients of the Child Psychiatry Department of the Regional Hospital in Kristiansand participated to this study. They were recently diagnosed as HKD F90.0 [12]. The boys in the ADHD group were all candidates for methylphenidate evaluation. Diagnostic assessment was carried out by a physician or a clinical psychologist, based on clinical interview and other sources of information available, including parent and school reports for the last 12 months, reports from other health professionals, and behavioral observations during the assessment period. Children were excluded if they met criteria for conduct- or oppositional defiant disorder, a depressive or anxiety disorder, Asperger's or Tourette's syndrome, or epilepsy. The group displayed full scale IQs within the normal range (Mean 97.6, SD 15.6), as assessed with the WISC-R [54].

Twenty-seven boys, all Caucasian Norwegians, aged 8–11 years (Mean age 9.5 SD 1.1) without ADHD were recruited from two local schools, one for young and one for older children, to the control group. The schools were located in a suburban area with a predominantly Caucasian, low middle class population with no known history of socio-cultural problems. Written permission was obtained from the Local Education Authority of the municipality. A letter with information about the project, and Barkley's DSM-IV rating scale of ADHD [55] was distributed by the school to parents of all boys age 8–11 years. An affirmative reply to participate was returned in a prepaid envelope addressed to the first author of this article. To exclude possible ADHD problems in the control group the children were rated with Barkley's DSM-IV rating scale of ADHD both by parents and teachers. Subjects were excluded from the control group if they met the DSM-IV criteria for ADHD at home or at school. The broader ADHD criteria (compared to HKD) were used to decrease the probability of including children with a high incidence of ADHD symptoms. No precautions were taken to exclude other clinical groups. Of at total of 35 subjects agreeing to participate, 8 were excluded from the study and referred to the local medical centre for further assessment of ADHD.

WISC-R testing was not carried out in the control group due to resource constraints. Time restrictions and the special recruitment procedures applied, involving only freshly diagnosed children, prohibited a close matching of age between the groups. This resulted in a slightly older ADHD group, difference between mean age = 0.7 years, p < .05. Matching of socio-economic background was not performed for the same reasons.

### The instrument

The MFNU was developed by the first author during the 1990-ies in close collaboration with well-educated and specialized personnel trained within the fields of ADHD, learning and behavioral problems. Some of the subtests were originally developed, and some are modified versions of subtests of the Danish 'Funksjonsnevrologisk Undersøgelse (FNU)' [56]. The subtests were primarily chosen and designed to reveal problems with motor inhibition and increased muscle tone.

In MFNU a qualitatively based scoring system is used. The test is performed in a very "dynamic" and interactional way with no limits concerning time and number of attempts in order to focus the attention of the child [1,9].

Most of the subtests of the MFNU (see Table 1) are performance tests where the child is given an instruction to perform a certain task (subtest 01–12). Subtests 13–16 are "passive" tasks where the tester evaluates muscular resistance while assessing the movement of the hips and feet. Item 17 'Synkinesis' is an evaluation of the presence of synkinetic movements during the examination. The subtest 'Palpation', which is included in the standard MFNU battery, was omitted from the study, as it can not be scored on the bases of video recordings. Therefore, only 17 subtests were used in the present study (see Additional files 3, 4, 5, 6, 7, 8, 9, 10, 11, 12, 13, 14, 15, 16).

A Cronbach's Alpha was calculated on the Total MFNU score in both groups. An Alpha of 0.98 indicates a very high relationship (internal consistency) within the total set of subtests [57] making the use of a total score for all variables meaningful, and supporting the assumption that the sum score may be treated as a continuous variable.

The MFNU is described in detail in a user manual and an accompanying DVD [9]. Table 1 presents a brief description of the subtests used, video films of the subtests are given in Additional Files 3 to 16.

MFNU is scored on a sheet applying a ranked 3-category format (0-1-2). The subtests are scored according to the criteria in Table 2. More detailed criteria for each subtest are presented in the MFNU manual and visualized in the accompanying DVD [9].

**Table 2:** Scoring criteria for the 17 subtests of MFNU

Score:		Criteria		
		subtests 01–12	subtests 13–16	subtest 17
0	'No problems'	The task is performed with no problems and little effort	Normal resistance against the movement is registered	Only sporadic synkinetic movements are registered
1	'Moderate problems'	The task is performed according to instruction, but with lot of attention and effort, or quality of performance is below what is expected for age	Resistance against the movement is registered	Moderate synkinetic movements are registered in one or more subtest
2	'Severe problems'	The child can not perform the task according to the instruction	Severe resistance against the movement is registered	Pronounced synkinetic movements are registered in one or more subtest

Normally when evaluating test-retest changes in performance with the MFNU a 7-category scoring system is used [9]. This system yields a more detailed picture of subtle changes in performance than the 3-category system. However, for the case of simplicity in the presentation of the results the basic 3-category scoring system was applied in the comparison of the two assessment sessions.

Preliminary inter-rater reliability analyses have shown high to very high degree of agreement among raters on all subtests (Cohen's Kappa ranging from 0.67 to 1.00) [1].

### Procedure

The present study involved two assessments with MFNU for all children 'Assessment 1' (A1) and 'Repeated assessment' (A2), with at an interval of at least 1 day (range 1–24 days, mean 4.54 days). The repeated assessment (A2) was performed in order to investigate possible training effects due to repeated testing. To prevent possible negative test results due to distraction or to emotional reactions to an unfamiliar situation, the ADHD group was given a preliminary session introducing the test situation and the different subtests to the parents and child. The assessment of the ADHD group took place at the hospital with the parents being present.

The control group was assessed at school. Practical restraints prohibited a preliminary consultation and participation of the parents during the assessment. Practical considerations and limited resources made it necessary to test the ADHD- and control group in different environments, thus prohibiting a blinded design. The subjects were assessed individually by a physiotherapist (the first author of this article) and videotaped by the physician (the fourth author). The videotapes were rated at a later point in time by a physiotherapist with no prior experience with the children. This physiotherapist had long experience both with administering the MFNU and with video rating MFNU sessions. To evaluate differences in performance between the two sessions, two parallel monitors were used. One scoring sheet was used for both assessments.

### Statistical analyses

The statistic analyses were carried out using SPSS software 15.0. Descriptive statistics were used on data from each subtest in the ADHD and control group in order to view the percent distribution of the scoring categories (0-1-2) and on the variable 'Total score' which is the sum of the scores on 17 subtests. 'Total score' ranged from 0 – 34. The non-parametric Mann-Whitney U-test was used to compare the ranked scores of the ADHD and control group on each of the 17 subtest. Since the data were not assumed to be normally distributed, the Mann-Whitney test was used to compare the ADHD- and control group on the 'Total score'. Pearsons Chi square test was used, due to the small group sizes, when examining possible effects of age on motor performance. A Cronbach alpha analysis was performed to establish the internal consistency of the total set of subtests. The Wilcoxon Signed Rank Test for related samples was used to compare changes in performance on repeated measurements.

### Approvals

The study was approved by The Norwegian Data Inspectorate, The Regional Committee for Medical Research Ethics, the research committee and the director of medicine at the Department of Child Psychiatry, Sørlandet Hospital, Kristiansand, Norway, and the school authorities at the municipality of Songdalen, Norway, where the control group was recruited.

## Results

The ADHD group showed a high percentage of 'severe problems' (score 2) in most of the subtests, most pronounced in the 'Passive abduction of hip' subtests (13–14) with a 'severe' score in 80–84% of the cases. When the 'moderate problems' and 'severe' scores (score 1 and 2) were combined, the ADHD group presented problems within a range of 80% ('Catch ball' and 'Walking') to 96% ('Dynamic balance, 1 leg' and 'Diadochokinesis, left'). The control group typically presented few, if any severe problems (see Table 3).

**Table 3:** The percent distribution of the ADHD- (N = 25) and Control group (N = 27) on the Assessment 1

	Score 0 %		Score 1 %		Score 2 %		Score 1 or 2 %	
Subtests of MFNU	ADHD	*Control*	ADHD	*Control*	ADHD	*Control*	ADHD	*Control*
01. Dynamic balance-2 legs	12.0	*77.8*	36.0	*22.2*	52.0	*0.0*	88.0	*22.2*
02. Dynamic balance-1 leg	4.0	*74.1*	28.0	*22.2*	68.0	*3.7*	96.0	*25.9*
03. Diadochokinesis-right	8.0	*70.4*	28.0	*14.8*	64.0	*14.8*	92.0	*29.0*
04. Diadochokinesis-left	4.0	*55.6*	32.0	*37.0*	64.0	*7.4*	96.0	*44.4*
05. Reciprocal coordination	12.0	*85.2*	28.0	*7.4*	60.0	*7.4*	88.0	*14.8*
06. Thumb movement	12.0	*81.5*	20.0	*18.5*	68.0	*0.0*	88.0	*18.5*
07. Throw ball	12.0	*85.2*	32.0	*14.8*	56.0	*0.0*	88.0	*14.8*
08. Catch ball	20.0	*66.7*	32.0	*18.5*	48.0	*14.8*	80.0	*33.3*
09. Walking	20.0	*77.8*	12.0	*22.2*	68.0	*0.0*	80.0	*22.2*
10. Lifting arm	8.0	*92.6*	36.0	*7.4*	58.0	*0.0*	92.0	*7.4*
11. Lifting leg	16.0	*88.9*	40.0	*11.1*	44.0	*0.0*	84.0	*11.1*
12. "Flying"	8.0	*85.2*	32.0	*14.8*	60.0	*0.0*	92.0	*14.8*
13. Passive abduction-r.hip	12.0	*92.6*	4.0	*7.4*	84.0	*0.0*	88.0	*7.4*
14. Passive abduction-l.hip	8.0	*92.6*	12.0	*7.4*	80.0	*0.0*	92.0	*7.4*
15. Passive movement-r.foot	8.0	*100.0*	20.0	*0.0*	72.0	*0.0*	92.0	*0.0*
16. Passive movement-l.foot	16.0	*88.9*	20.0	*11.1*	64.0	*0.0*	84.0	*11.1*
17. Synkinesis	12.0	*66.7*	24.0	*29.6*	64.0	*3.7*	88.0	*33.3*

Score 0: 'No problems'; Score 1: 'Moderate problems'; Score 2: 'Severe problems'

The Mann-Whitney U-test showed highly significant differences between the groups on all 17 subtests both on the A1 and the A2. The subtest 'Catch ball' showed the lowest p-value (p < .005), while the rest of the subtests showed p < .001. The differences were most pronounced in the youngest children (8–10 years). However, significant differences were also obtained between the older children with ADHD and the control group on all the subtests except for 'Catch ball' (see Table 4).

**Table 4:** The results of Mann-Whitney U-tests on the subtests of MFNU on Assessment 1

	**The entire sample** ADHD gr.(N = 25 age 8–12) Contr. gr (N = 27 age 8–11)			**Age < 11 years** ADHD gr (N = 12) Contr. gr (N = 20)			**Age 11–12 years** ADHD gr (N = 13) Contr. gr (N = 7)		
Subtests of MFNU	Mann-Whitney U	z-value	Asymp. Sig. (2-tailed).	Mann-Whitney U	z-value	Asymp. Sig. (2-tailed)	Mann-Whitney U	z-value	Asymp. Sig. (2-tailed)
01. Dynamic balance-2 legs	76.5	-5.2	.000	16.0	-4.5	.000	16.0	-2.5	.012
02. Dynamic balance-1 leg	53.5	-5.6	.000	11.0	-4.6	.000	9.5	-3.0	.002
03. Diadocho- kinesis-right	109.0	-4.5	.000	24.0	-4.1	.000	19.5	-2.2	.029
04. Diadocho- kinesis-left	91.5	-5.2	.000	20.0	-4.2	.000	17.5	-2.4	.016
05. Reciprocal coordination.	82.5	-5.1	.000	34.0	-3.8	.000	3.5	-3.6	.000
06. Thumb movement	60.5	-5.5	.000	4.0	-4.9	.000	13.5	-2.7	.006
07. Throw ball	62.5	-5.5	.000	16.0	-4.5	.000	10.5	-3.0	.003
08. Catch ball	166.0	-3.4	.001	47.5	-3.0	.002	27.5	-1.5	.125
09. Walking	91.5	-4.9	.000	9.0	-4.6	.000	17.5	-2.6	.009
10. Lifting arm	38.0	-4.8	.000	4.0	-5.1	.000	7.0	-3.3	.001
11. Lifting leg	75.0	-6.0	.000	20.5	-4.3	.000	10.5	-3.0	.003
12. "Flying"	47.0	-5.3	.000	6.0	-4.8	.000	7.0	-3.3	.001
13. Passive abduct-right hip	44.5	-5.8	.000	.0	-5.3	.000	10.5	-3.1	.002
14. Passive abduct-left hip	32.0	-6.1	.000	1.0	-5.2	.000	7.0	-3.4	.001
15. Pas. move-ment-right foot	27.0	-6.3	.000	.0	-5.5	.000	7.0	-3.3	.001
16. Pas. move-ment-left foot	67.5	-6.4	.000	3.0	-5.1	.000	17.0	-2.5	.013
17. Synkinesis	92.0	-5.5	.000	15.0	-4.3	.000	10.5	-3.0	.003

Total score	29.5	-5.7	.000	1.0	-4.7	.000	4.5	-3.3	.001

When split into two age groups (12 subjects were < 11 and 13 were > = 11 years) the older ADHD group performed significantly better than the younger group on two subtests, 'Reciprocal coordination' and 'Walking' (p < .05 using Pearsons Chi square test).

Table 5 presents the statistics and central tendencies for the 'Total score' in the two groups on the A1 and the A2 sessions. This difference between the groups was particularly reflected in median scores.

**Table 5:** Statistics for ADHD and control group on assessment 1 and repeated assessment on the 'Total score'

	'Total score' Assessment 1		'Total score' Assessment 2	
	ADHD	Control	ADHD	Control
N	25	27	24	27
Range	33	20	22	20
Min	1	0	12	0
Max	34	20	34	20
Median	28	1	29	1

p < .0001 on both trials was found comparing the 'Total score' scores for the ADHD and control group (Mann-Whitney U-test).

**Figure 1:**
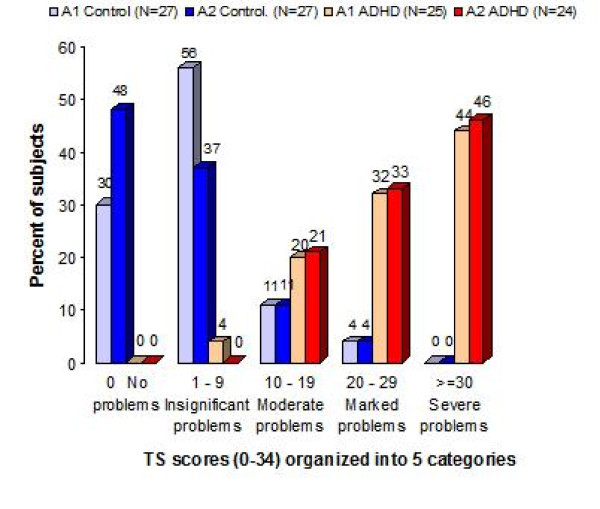
**Distribution of TS scores for the Assessment 1 and Assessment 2**. The percent distribution of the 'Total score'. The graph presents the distribution of the 'Total scores' for the ADHD and the non-ADHD control group on assessment 1 (ADHD group N = 25; control group N = 27) and assessment 2 (ADHD group N = 24; control group N = 27), with the 'Total score' scores ranging from 0–34, grouped into 5 intervals.

Figure 1 displays the 'Total score' for the ADHD and control group on A1 and on A2.

Mann-Whitney U-tests showed that the ADHD group (Median = 28) had significantly more motor problems (higher 'Total score') than the control group (Median = 1) both on A1: U = 26.6, p < .0001, and on A2: ADHD group (Median = 29), control group (Median = 1): U = 8.5, p < .0001. Cohen's δ of the 'Total score' between the groups on A1 was 1.67.

Wilcoxon signed ranks tests were performed on each subtest score and 'Total score' of the A1 and the A2. No significant improvement was obtained on individual subtests or for the 'Total score' within the ADHD group. For the control group a minor, but significant improvement was found for the 'Total score' (p < .05). Motor problems in the control group were most frequent on the subtests '07 Catch ball' and on '03–04 Diadochokinesis', and least frequent on the 'Passive movement' (13–16) and on the 'Extension' subtests (10–12). In the control group, the two subjects with the highest 'Total score' score (14 and 20), both referred for physiotherapy after the study, showed little or no problems on the passive movement subtests and only moderate problems on the extension subtests. In the ADHD group the strongest differences from the control group were found on the 'Passive movement' and 'Extension' subtests.

## Discussion

Our hypothesis that there is a discriminative power of the MFNU between boys aged 8–12 years with ADHD (HKD F90.0) and controls without ADHD was strongly supported by the test data across all subtests. Most of the ADHD-subjects achieved a marked to severe 'Total score'. While there were subjects in the control group who showed problems on some of the subtests, the problems appeared on fewer subtests and with less severity than in children in the ADHD group. There were no significant improvements in performance from A1 to A2 within the ADHD group. The high incidence of motor problems within the ADHD group, on all subtests of the MFNU, and the lack of training effect from repeated exposures to the subtests was consistent with our previous clinical observations.

The passive movement subtests (13–16) discriminated strongly between the groups. These results seem to confirm our clinical observations that high muscle tone and neuromuscular inhibition problems seen in ADHD are not associated with inattention or impulsivity. Problems involved in the passive movement- and extension tests are not part of the criteria for DCD. These subtests were specifically designed for the MFNU, and are not included in standard motor assessment batteries. On the item 'Passive movement of the right foot' 100% of the controls showed no restriction against the passive movement, whereas 92% of ADHD group had a score of 1 or 2 on this item.

We found that motor problems are present in a higher percentage in the ADHD group (see Table 3) than the around 50% reported in previous studies [18,58]. At present there is no "gold standard" for testing motor problems in children [59,60]. Different instruments may measure motor functions that are not typically seen in ADHD, or may be insensitive to significant qualitative aspects of motor problems in this condition. In the MFNU *repeated movements *are emphasized in many subtests in order to reveal *increasing *inhibition problems when the movements are performed repeatedly. These aspects of motor inhibition problems are usually not assessed in traditional standardised tests, increasing the likelihood of overlooking significant problems.

Another reason for the variations in reported motor problems could be related to diagnostic practice, with consequences for the selection of ADHD-samples. Much criticism has been raised against the DSM-IV definition of ADHD for the inclusion of clinical groups that may have different problems as the base of their overt behaviour [51,61]. When investigating motor problems in ADHD, this diagnostic practice may seriously confound the results, leading to premature conclusions that motor problems in ADHD are less frequent than shown in the present study. Our ADHD sample was limited to boys with HKD F90.0 [11,12] in the age range of 8–12 years. By limiting the sample in this way, the probability of including misdiagnosed children (false positives) in the ADHD group, and undiagnosed children with ADHD (false negatives) in the control group was highly reduced. We therefore find it improbable that the high occurrence and severity of motor problems in the ADHD group found in this study, was due to a biased sample.

## Limitations

Some of the children with ADHD probably met the criteria for the DCD diagnosis, even if none of them had been referred for motor problems. One might argue that these subjects should have been excluded from the sample in order to get a purest possible ADHD sample. We recommend that this is done in futures studies. Furthermore, the introduction of a control group consisting of DCD subjects, without ADHD, could have contributed to illustrate to what extent the motor problems of the ADHD children were specifically associated with ADHD and not DCD. There is also a need to investigate the possible overlap of motor problems in ADHD with other clinical groups. This could contribute to decide whether the motor problems seen in ADHD are unique and specific to the disorder.

Another issue that needs to be clarified is related to the design of the study. The ADHD group and the control group were assessed at different locations. The fact that the rater knew the group membership may have yielded a biased scoring. However, a more blinded design would probably not have eliminated rater bias. Our experienced rater would have guessed the group-membership of the children with ADHD from their restless and impulsive behavior, even if the children were assessed in a randomized and blinded design. While experimenter bias due to prejudiced rater expectations may have contributed to a falsely high difference between the ADHD- and control group, the very significant differences shown in our study rule out the possibility that bias rating alone could explain the results. The high inter tester-reliability of the MFNU further supports this conclusion.

The children in the ADHD group were assessed with parents attending and they also had a preliminary trial session. It is unlikely that these facts had any effect on the groups that could weaken the differences in performance. On the contrary, *less *motor problems in the control group might have been expected if these children had been allowed a preliminary session.

The 3-category scoring system applied in this study has been evaluated as reliable in clinical practice [1], and is fairly easy to administer. For the purposes of our present study the 3-category scoring system has proven satisfactory. However, for future research purposes, a more differentiated scaling system would be preferable. This would also facilitate statistical analyses, particularly factor analyses of possible subscales that may differentiate between clinical groups. We are presently considering alternative scoring systems that offer a fair compromise between reliability considerations and the need for precision.

## Conclusion

This study has demonstrated that the MFNU is a sensitive instrument for assessment of functional problems in boys with HKD associated with motor inhibition and stability. It also strongly indicates that these motor problems are present in a very high percentage of children with ADHD. Future studies are needed to confirm these findings and also to investigate to what extent the same problems are present in girls, and in older persons with HKD. While a high total score on the MFNU may be strongly correlated with the HKD diagnosis, challenging the present assumption that motor problems are not central to the core functional problems of the condition, our study provides no evidence that a high MFNU-score can be used as an indicator of ADHD. Even if the MFNU is promising as a diagnostic tool, there is still a way to go to clarify the differentiating power of the instrument when used in connection with all the DSM-IV subgroups of ADHD, and with other clinical groups.

## Competing interests

The MFNU manual is sold by the Reading centre, University of Stavanger for kr 700 (about €85). LLS receive 9%, TS 3% and SI 3% royalties.

## Authors' contributions

LLS has been the main contributor both in conception, design, acquisition, analysis and interpretation of data. She also is the main contributor in the drafting of the manuscript.

TS has been involved in the analysis and interpretation of data, and in the drafting and revision of the manuscript.

SI has been a contributor in the conception, design and revision of the manuscript.

AR has been a contributor to the conception, design and acquisition of data.

BE has contributed to the conception, design, interpretation of data and in revising the drafts of the manuscripts. He has given final approval of the version to be published.

FET has been involved in the data analysis and in final revision and approval of the manuscript.

## Supplementary Material

Additional File 1 **Thumb movement ADHD no medication**. This movie shows the MFNU subtest 'Thumb movement' performed by a child diagnosed with ADHD-C/HKD. The child had not received MPH the current day or the day before. The move is from the DVD accompanying the MFNU manual [9]. Click here for file

Additional File 2 **Thumb movement ADHD with MPH**. This movie shows the MFNU subtest 'Thumb movement' performed by a child diagnosed with ADHD-C/HKD. The movie was made 1 1/2 after medication with 10. mg MPH on the same day as the previous movie. The move is from the DVD accompanying the MFNU manual [9]. Click here for file

Additional File 3 **01_Dynamic balance – 2 legs**. This movie shows the MFNU subtest 01 'Dynamic balance – 2 legs' performed by a child without ADHD or motor problems. The move is from the DVD accompanying the MFNU manual [9]. Click here for file

Additional File 4 **02_Dynamic balance – 1 leg**. This movie shows the MFNU subtest 02 'Dynamic balance – 1 leg' performed by a child without ADHD or motor problems. The movie is from the DVD accompanying the MFNU manual [9]. Click here for file

Additional File 5 **03 and 04_Diadochokinesis – right and left**. This movie shows the MFNU subtests 03 and 04 'Diadochokinesis – right' and 'Diadochokinesis – left' performed by a child without ADHD or motor problems. The move is from the DVD accompanying the MFNU manual [9]. Click here for file

Additional File 6 **05_Reciprocal coordination**. This movie shows the MFNU subtest 05 'Reciprocal coordination' performed by a child without ADHD or motor problems. The move is from the DVD accompanying the MFNU manual [9]. Click here for file

Additional File 7 **06_Thumb movement**. This movie shows the MFNU subtest 06 'Thumb movement' performed by a child without ADHD or motor problems. The movie is from the DVD accompanying the MFNU manual [9]. Click here for file

Additional File 8 **07_Throw ball**. This movie shows the MFNU subtest 07 'Throw ball' performed by a child without ADHD or motor problems. The movie is from the DVD accompanying the MFNU manual [9]. Click here for file

Additional File 9 **08_Catch ball**. This movie shows the MFNU subtest 08 'Catch ball' performed by a child without ADHD or motor problems. The movie is from the DVD accompanying the MFNU manual [9]. Click here for file

Additional File 10 **09_Walking**. This movie shows the MFNU subtest 09 'Walking' performed by a child without ADHD or motor problems. The movie is from the DVD accompanying the MFNU manual [9]. Click here for file

Additional File 11 **10_Lifting arm**. This movie shows the MFNU subtest 10 'Lifting arm' performed by a child without ADHD or motor problems. The movie is from the DVD accompanying the MFNU manual [9]. Click here for file

Additional File 12 **11_Lifting leg**. This movie shows the MFNU subtest 11 'Lifting leg' performed by a child without ADHD or motor problems. The movie is from the DVD accompanying the MFNU manual [9]. Click here for file

Additional File 13 **12_Flying**. This movie shows the MFNU subtest 12 'Flying' performed by a child without ADHD or motor problems. The movie is from the DVD accompanying the MFNU manual [9]. Click here for file

Additional File 14 **13 and 14_Passive abduction – right and left hip**. This movie shows the MFNU subtests 13 and 14 'Passive abduction – right hip' and Passive abduction – left hip' performed by a child without ADHD or motor problems. The movie is from the DVD accompanying the MFNU manual [9]. Click here for file

Additional File 15 **15 and 16_ Passive movement – right foot and left foot**. This movie shows the MFNU subtests 15 and 16 'Passive movement – right foot and left foot' performed by a child without ADHD or motor problems. The movie is from the DVD accompanying the MFNU manual [9]. Click here for file

Additional File 16 **17_Synkinesis**. This movie shows the MFNU subtest 17 'Synkinesis'performed by a child without ADHD or motor problems. The movie is from the DVD accompanying the MFNU manual [9]. Click here for file
